# Pleural and Pericardial Effusions Associated with Semaglutide: A Case Report

**DOI:** 10.5811/cpcem.48864

**Published:** 2026-01-21

**Authors:** Maggie Stark, Nicholas Valentini

**Affiliations:** Brown University, Department of Emergency Medicine, Providence, Rhode Island

**Keywords:** semaglutide, pleural effusions, drug-induced lupus, case report

## Abstract

**Introduction:**

Semaglutide, a glucagon-like peptide-1 receptor agonist, has gained increasing popularity for managing both type 2 diabetes mellitus and obesity. However, as its use increases, new adverse events are emerging. This case report presents a 70-year-old patient who developed pleural and pericardial effusions likely related to semaglutide use.

**Case Report:**

Four weeks after being prescribed semaglutide, a 70-year-old woman presented to the emergency department (ED) with shortness of breath. Diagnostic testing in the ED and hospital revealed that she had both pericardial and exudative pleural effusions, along with a positive anti-nuclear antibody and elevated inflammatory markers. Her signs and symptoms improved with steroid administration, and no other etiology was identified.

**Conclusion:**

The patient was diagnosed with drug-induced lupus, likely triggered by semaglutide. This case underscores the importance of recognizing and investigating uncommon drug-related complications. With the growing use of semaglutide, clinicians must remain vigilant for rare adverse effects such as pleural effusions to ensure prompt diagnosis and treatment.

## INTRODUCTION

Obesity has become an increasing problem within the United States. Due to the significant comorbidities associated with obesity, pharmaceutical solutions have been in great demand. Semaglutide, a glucagon-like peptide-1 (GLP-1) receptor agonist, has gained increasing popularity for managing both type 2 diabetes mellitus and obesity. It has been shown to be effective for weight loss and improving cardiometabolic risk factors when compared to a placebo.^1^ It is known to most commonly cause gastrointestinal side effects^1^; however, as its use increases, new adverse events are emerging. In this case report we discuss a patient who developed pleural and pericardial effusions likely related to semaglutide use.

## CASE REPORT

A 70-year-old woman with a past medical history significant for hypertension, hyperlipidemia, Raynaud phenomenon, and seasonal allergies presented to the emergency department (ED) for shortness of breath and chest tightness. Her symptoms had progressively worsened over three weeks. She had started semaglutide six weeks prior for weight loss and began experiencing nausea, shortness of breath, and chest discomfort three weeks after initiation. She was advised to discontinue the medication in week four, but her symptoms persisted, prompting an ED visit.

On physical exam, her vitals were as follows: oxygen saturation, 95%; heart rate, 99 beats per minute; blood pressure, 127/63 millimeters of mercury; respiratory rate, 18 breaths per minute; and temperature, 36.7 °C. Physical exam was notable for pitting edema to the lower extremities, left greater than right, and coarse rales at the bilateral lung bases. A complete blood count, complete metabolic panel, brain natriuretic peptide (BNP), D-dimer, electrocardiogram, troponin assay, and chest radiograph (CXR) were ordered. Ultrasonography was performed including a lower extremity vascular ultrasound of the left lower extremity and a point-of-care ultrasound of the heart and lungs. The vascular ultrasound showed no evidence of deep vein thrombosis in the lower extremities.

**Image 1:**
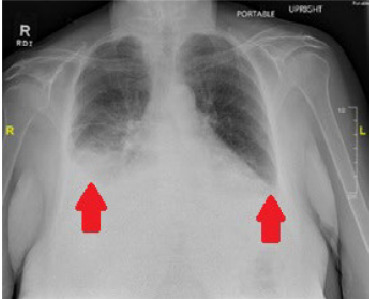
Chest radiograph showing bilateral pleural effusions greater on the right than the left. Arrows point to bilateral pleural effusions.

**Image 2:**
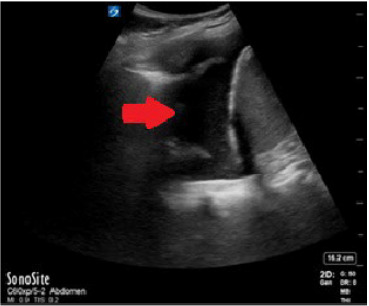
Point-of-care thoracic ultrasound showing a right-sided pleural effusion with the diaphragm in view. Arrow indicates pleural effusion.

**Image 3:**
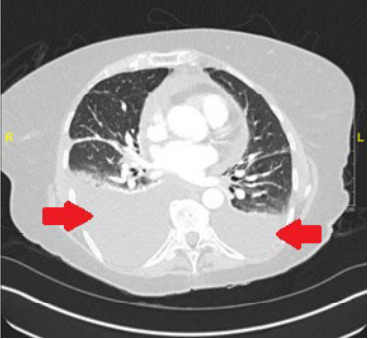
Computed tomography angiography of the chest showing bilateral pleural effusions, greater on the right than the left. Arrows point to bilateral pleural effusions.

Her point-of-care echocardiography demonstrated a small, circumferential pericardial effusion without evidence of decreased ejection fraction and without right heart strain. Her CXR and point-of-care lung ultrasound demonstrated bilateral pleural effusions, more significant on the right (Images 1 and 2). Her D-dimer was elevated at 1,102 nanograms per milliliter (ng/mL) (reference range < 230 ng/mL). Subsequent computed tomography (CT) angiography of the chest did not demonstrate any pulmonary embolism; however, it did show moderate bilateral pleural effusions greater on the right (Image 3). The remainder of her lab testing was within normal limits.

Rheumatology was consulted with concern for an autoimmune process contributing to her pleural effusions. Rheumatology recommended that additional labs be collected. Pertinent results included an elevated C-reactive protein (CRP) at 110.9 milligrams per liter (mg/L) (< 10.0 mg/L) and an erythrocyte sedimentation rate at 66 mm/hour (0-30 mm/hour). She had a positive anti-nuclear antibody (ANA) with a speckled pattern and 1:160 titer (< 1:40) and anti-cyclic citrullinated peptide (anti-CCP) antibody level of 131 units (< 20 units). Additional autoimmune studies (anti-double stranded DNA, anti-histone, anti-Smith, anti-Ro antibody, anti-La antibody, complement 3, complement 4, and rheumatoid factor) were negative. The patient was then admitted to the hospital on the internal medicine service for further testing.

*CPC-EM Capsule*

What do we already know about this clinical entity?

*Semaglutide, a glucagon-like peptide-1 agonist, is effective for weight loss, but the spectrum of side effects is still being discovered.*

What makes this presentation of disease reportable?

*A patient who developed pleural and pericardial effusions after semaglutide use was diagnosed with drug-induced lupus despite negative anti-histone antibodies.*

What is the major learning point?

*Semaglutide has a developing side-effect profile, including rarely seen pathology such as drug-induced lupus. Point-of-care ultrasound can aid in rapid diagnosis.*

How might this improve emergency medicine practice?

*Awareness of atypical semaglutide side effects helps clinicians broaden differentials and identify serious complications early.*

While admitted, the patient underwent a diagnostic thoracentesis with fluid studies suggestive of an exudative process. Her serum protein measured 6.3 grams (g)/dL (6.1–8.3 g/dL) and her pleural fluid protein measured 4.2 g/dL. Her serum lactate dehydrogenase (LDH) measured 217 IU/L (119–265 IU/L) while her pleural fluid LDH measured 145 IU/L. Cytology of the pleural fluid showed mixed, non-specific inflammatory changes without evidence of malignancy. Culture of the pleural fluid including bacterial, fungal, and acid-fast bacilli did not produce any growth suggesting against an infectious etiology. Cardiac studies including a cardiology-based echocardiogram did not show any evidence of heart failure. Cardiology specialty consultation did not believe her pleural effusions were related to structural heart disease or heart failure. Infectious disease was also consulted and did not believe her pleural effusions originated from an infectious etiology.

Ultimately, following multispecialty discussions, the patient was placed on a prolonged oral steroid taper with a provisional diagnosis of drug-induced lupus despite negative anti-histone antibodies. At outpatient follow-up three months after hospitalization with rheumatology, the patient had marked improvement in symptoms with resolution of the pleural and pericardial effusions on follow-up imaging. She continued to do well from a clinical perspective.

## DISCUSSION

Atraumatic pleural effusions may be caused by several different clinical entities such as heart failure, parapneumonic effusion, cirrhosis, infection, and pulmonary embolism (PE) with less common etiologies including malignancy, renal disease, and drug-induced and autoimmune factors. In this case, a 70-year-old woman presented with symptoms of shortness of breath, chest tightness, and nausea, which were temporally associated with her initiation of semaglutide. The evaluation of pleural effusions, along with her clinical course and lab results, required a systematic approach to rule out various potential causes. She had no recent history of infection, antibiotic use, fever, travel, cough or associated symptoms, making an infectious cause of her pleural effusion less likely. Pleural fluid cultures did grow any pertinent organisms.

Given her age and risk factors, including hypertension, new-onset heart failure was initially considered high on the differential diagnosis, although this was not supported by subsequent point-of-care ultrasound and lab evaluation, namely a finding of a preserved ejection fraction and low BNP. A confirmatory cardiology-based echocardiogram performed later in the patient’s hospital course was also normal. Given the shortness of breath, PE and resultant reactive effusion was also considered. A D-dimer was elevated prompting a CT demonstrating the bilateral pleural effusions without evidence of PE.

The temporal relationship between the initiation of semaglutide and the onset of the patient’s symptoms raised suspicion for drug-induced lupus. Semaglutide has been associated with immune-mediated side effects, including lupus-like reactions.^2^,^3^ While the patient did not present with the classic symptoms of lupus, such as a butterfly-shaped rash or photosensitivity, the positive ANA and anti-CCP antibodies, along with a raised CRP, were indicative of an inflammatory process. By the Light criteria, the pleural fluid protein and LDH values have a 98% sensitivity and 83% specificity for exudative physiology.^4^ The exudative nature of the pleural effusions and the negative workup for other autoimmune diseases (eg, negative anti-dsDNA, anti-Smith antibodies) further supported this diagnosis. The patient’s improvement after stopping the offending medication, a prolonged steroid course, and supported by follow-up imaging and clinic visits reinforced the diagnosis.

Drug-induced lupus presents without anti-histone antibodies in approximately 25% of cases.^5^,^6^

There is emerging evidence that newer medications have a lower association with anti-histone antibody production despite the same clinical presentation.^7^ Semaglutide, a new medication widely prescribed for weight loss, has become increasingly popular. A 2022 meta-analysis reported a two-fold likelihood ratio of adverse events in patients taking semaglutide when compared to placebo.^8^ Patients were also 1.6 times more likely to experience severe adverse outcomes including death and prolonged hospitalization.^7^ Drug-induced lupus from semaglutide has been previously reported, although it was associated with a positive anti-histone antibody test.^9^ However, the patient presented with symptoms of acute liver failure and autoimmune hepatitis with intra-abdominal ascites rather than the primary thoracic pathology identified in this case report.^9^

## CONCLUSION

More study is needed to establish a definitive association between semaglutide and the autoimmune phenomena described. This case highlights the need to consider atypical causes of pleural effusion, especially in patients with recent medication changes, and to remain vigilant for rare adverse effects that can mimic more common pathologies.

## FINAL DIAGNOSIS

Exudative pleural and pericardial effusions associated with semaglutide.

## References

[1] Amaro A, Sugimoto D, Wharton S (2022). Efficacy and safety of semaglutide for weight management: evidence from the STEP program. Postgrad Med.

[2] Rivera FB, Arias-Aguirre E, Aguirre Z (2024). Evaluating the safety profile of semaglutide: an updated meta-analysis. Curr Med Res Opin.

[3] Ruder K (2023). As semaglutide’s popularity soars, rare but serious adverse effects are emerging. JAMA.

[4] Light RW, Macgregor MI, Luchsinger PC (1972). Pleural effusions: the diagnostic separation of transudates and exudates. Ann Intern Med.

[5] Epstein A, Barland P (1985). The diagnostic value of antihistone antibodies in drug-induced lupus erythematosus. Arthritis Rheum.

[6] Solhjoo M, Goyal A, Saliba F (2023). Drug-induced lupus erythematosus. StatPearls.

[7] Lee AYS (2022). Clinical use of anti-histone antibodies in idiopathic and drug-induced lupus. Immunol Med.

[8] Tan HC, Dampil OA, Marquez MM (2022). Efficacy and safety of semaglutide for weight loss in obesity without diabetes: a systematic review and meta-analysis. J ASEAN Fed Endocr Soc.

[9] Castellanos V, Workneh H, Malik A (2024). Semaglutide-induced lupus erythematosus with multiorgan involvement. Cureus.

